# Design Optimization of a Gas Turbine Engine for Marine Applications: Off-Design Performance and Control System Considerations

**DOI:** 10.3390/e24121729

**Published:** 2022-11-25

**Authors:** Affiani Machmudah, Tamiru Alemu Lemma, Mahmud Iwan Solihin, Yusron Feriadi, Armin Rajabi, Mohamad Imam Afandi, Aijaz Abbasi

**Affiliations:** 1 Industrial Engineering, Faculty of Advance Technology and Multidisciplinary, Universitas Airlangga, Kampus C Jalan Mulyorejo, Surabaya 60115, Indonesia; 2 Research Center for Hydrodynamics Technology, National Research and Innovation Agency (BRIN), Jl. Hidro Dinamika, Keputih, Sukolilo, Surabaya 60112, Indonesia; 3 Department of Mechanical Engineering, Universiti Teknologi PETRONAS, Seri Iskandar 32610, Malaysia; 4 Faculty of Engineering, Technology and Built Environment, UCSI University, Kuala Lumpur 56000, Malaysia; 5 Department of Physics, Faculty of Mathematics and Natural Sciences, Institut Teknologi Bandung, Jl. Ganesha No 10, Bandung 40132, Indonesia; 6 Department of Mechanical and Manufacturing Engineering, Faculty of Engineering and Built Environment, Universiti Kebangsaan Malaysia, Bangi 43600, Malaysia; 7 Mechanical Engineering, Quaid-e-Awam University of Engineering, Science, and Technology, Nawabshah 67480, Pakistan

**Keywords:** gas turbine, control system, thermodynamics-based model, gain scheduling optimization, whale optimization algorithm, genetic algorithm, energy efficiency, marine propulsions

## Abstract

This paper addresses a design optimization of a gas turbine (GT) for marine applications. A gain-scheduling method incorporating a meta-heuristic optimization is proposed to optimize a thermodynamics-based model of a small GT engine. A comprehensive control system consisting of a proportional integral (PI) controller with additional proportional gains, gain scheduling, and a min-max controller is developed. The modeling of gains as a function of plant variables is presented. Meta-heuristic optimizations, namely a genetic algorithm (GA) and a whale optimization algorithm (WOA), are applied to optimize the designed control system. The results show that the WOA has better performance than that of the GA, where the WOA exhibits the minimum fitness value. Compared to the unoptimized gain, the time to reach the target of the power lever angle is significantly reduced. Optimal gain scheduling shows a stable response compared with a fixed gain, which can have oscillation effects as a controller responds. An effect of using bioethanol as a fuel has been observed. It shows that for the same input parameters of the GT dynamics model, the fuel flow increases significantly, as compared with diesel fuel, because of its low bioethanol heating value. Thus, a significant increase occurs only at the gain that depends on the fuel flow.

## 1. Introduction

Achieving energy efficiency has become a global issue. Small scale gas turbines have become of great interest, and their possible applications have been explored, especially in terms of achieving a sustainable energy power system. Market studies show that the demand for a small size gas turbine (GT) is increasing in contrast to larger scale GT [1]. The adaptability and low emission level of the small gas turbine are some of the benefits that are increasing their use [1,2]. In line with energy issues, the thermal power plant systems have exhibited greenhouse gas emissions, and technologies to reduce these bad environment effects are of great importance. For marine transportation, the International Maritime Organization (IMO) has created various regulations to control sulfur oxide (SOx) and nitrogen oxides (NOx) emissions and to increase ship energy efficiency [3]. Starting in 2020, a new IMO regulation began requiring a reduction in the SOx emission for shipping fuel from 3.5% to 0.5% (5000 ppm) [4].

The research presented in this paper aims to support the IMO goal of a clean environment to reduce the gas emissions created by marine transportation. Considerable efforts toward achieving the IMO clean energy goal have been made; however, it is still a challenge to convert the fossil fuel marine power system to a non-fossil fuel marine engine. Fossil fuel is known to be the main factor responsible for polluting the air and the environment.

One methodology to improve energy efficiency for offshore oil and gas platforms, which has made significant contributions in reducing greenhouse gas emissions, is the downsizing of the GT as the power system [5]. A smaller size GT power plant is less complex, more cost optimal, and easy to maintain [6]. Furthermore, the small/micro-GT is very adaptable to renewable energy fuels [7]. Thus, the GT becomes a potential candidate as marine power to achieve the goal of the clean technology of marine transportation. Studies to investigate the performance of the GT engine for marine applications have been conducted. Assessments of the use of mini gas turbines for naval transportation were conducted for the GT, with a power output range between 1 MW to 10 MW [8]. The small GT shows competitive cogeneration performance if compared to commercial GT and marine diesel engines. The mini/micro-GT is an excellent candidate for renewable energy systems because of its flexibility regarding various type of fuels, including biofuels [7]. Recent research has reported that biofuel can be a potential candidate for local or domestic sea shipping, depending on the availability of the biofuel resource [3]. However, implementing biofuel to the GT engine still poses some challenges. Some modification of the fuel delivery system generally is necessary, mainly due to their low heating values and high viscosity [9].

The objective of the present paper is to design the optimal comprehensive control system and stay within its operational area for marine applications of the small GT engine. For achieving the goal of clean energy for a marine power system, a study of the GT control system that is optimal and well-performing, at least satisfying the maximum ppm level of SOx when the fuel is renewable energy, is necessary.

**Figure 1:**
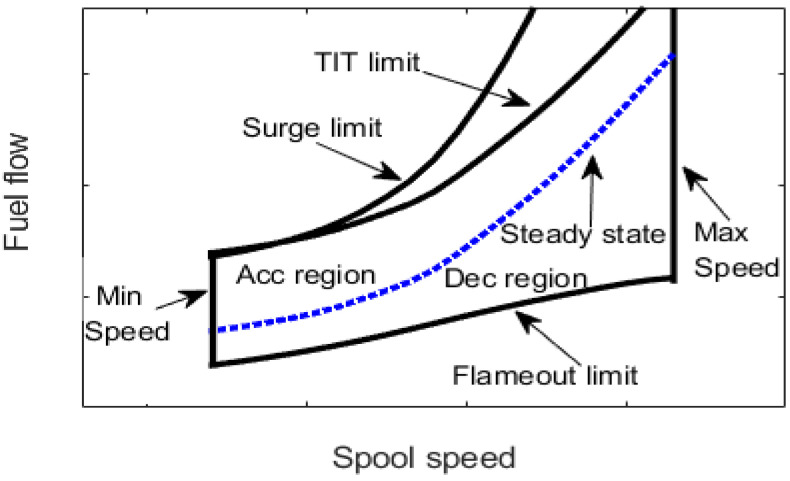
GT fuel boundary graph.

The GT engine has an operational range because of the performance limiters that construct the fuel boundaries. These limiters include the surge, turbine inlet temperature (TIT), minimum spool speed, maximum spool speed, and flameout limits. Figure 1 illustrates the typical GT fuel boundary [10]. The control system of the GT engine should facilitate the engine operation within this fuel boundary to ensure engine safety.

The dynamic model of the GT is required to obtain the controller performance. Kreiner and Lietzau [11] divide GT models into two base models, namely the thermodynamics model and the state space model. The state space model has the advantage of low computational demand; however, this method has less flexibility as compared with the thermodynamics-based model. The thermodynamics-based model can be used as a tool to design a new GT engine and to obtain off-design performance, as this model provides more detailed simulations, enhances the knowledge of engine working mechanism, and supports the engine system design. Moreover, detailed performance, as well as engine limiters, can be obtained and visualized to maintain the engine operation in the safe zone when the control system is designed within this permissible zone.

The gain scheduling technique was first applied to flight control and aerospace systems. Using the gain scheduling, the controller parameters are varied depending on the operating conditions. It is highly effective to control the system whose dynamics change with operating conditions, as in the GT. The previous studies mainly apply the gain scheduling technique to the state space-based model of the GT engine. In the engine fuel system application, J. G. Rivard [12] was the first to employ the gain scheduling technique to the automobile engine. Qi et al. [13] designed and evaluated the gain scheduling controller of a single-shaft GT engine. The requirements of a quick thrust response, considering the engine operational limits, were converted to the constraints in the form of the compressor characteristics. Garg [14] presented simplified scheduling scheme that explored the robustness of a multivariable control design. The optimization formulation for analysis of the scheduled gain was discussed. The proposed approach was applied to the turbofan engine for a short take-off and vertical landing (STOVL) airplane.

The LPV-based control techniques have been studied to synthesize the gain-scheduled controller of a turbofan engine [15,16]. Zhao et al. [17] proposed an approximate nonlinear model, namely an equilibrium manifold expansion (EME) model, derived from the linearization approach of a nonlinear system with operating points mapping. Pakhmer et al. [18] presented the stability and verification model of the gain scheduling of the GT engine control system. Using the global linearization and linear matrix inequality, the stability of the proposed approach was proved.

Huang et al. [19] proposed the gain scheduling for explicit model predictive control for a diesel engine air path. The optimizations of a quadratic programming problem were solved using Newton’s method and a nonlinear optimizer in MATLAB’s fmincon. Liu et al. [20] presented an improved version of the gain scheduling technique which can achieve the expected performance and stability for an aero-engine. The proposed method involved the scheduling parameters variation. Gou et al. [21] proposed a robust gain-scheduling using the estimation of the engine performance degradation. Robust controllers considering the normal engine operation and performance degradation were constructed at the operating conditions set. The controllers’ schedules were designed based on the appropriate scheduling and health of the parameters.

Pang et al. [22] developed a novel direct thrust control using an improved model predictive control via a strategy to reduce the control sequence dimension. The computation burden was significantly reduced by redefining the control sequence to optimize a single variable of control at each simulation time. The average time consumption was reduced by up to 65% that of the standard predictive control model.

Eslami and Banazadeh [23] addressed the problem of severe performance loss at the switching of the controller command. They proposed new heuristic approaches, namely α-method, ζ–method, and ϵ-method, to enhance the performance of the GT minimum command selection (MCS) control technique. The results showed that the proposed method upgraded the GT performance as compare with the traditional MCS and min-max technique.

Chen et al. [24] proposed a new direct performance adaptive predictive control based on a subspace-based improved model predictive control (SIMPC) to obtain the predictive control applicable for any engine range operation points. The proposed method reduced the calculation amount and ensured the estimation accuracy within their allowable operating points. The results demonstrated that the performance of the engine was fully explored by the optimization of the SIMPC instead of the min/max limit controller.

Shuwei et al. [25] developed a novel direct thrust control using an improved model predictive control via a strategy to reduce the control sequence dimension. The computation burden was significantly reduced by redefining the control sequence to optimize a single variable of control at each simulation time. The average time consumption was reduced up to 65% over the standard predictive control model.

The above previous studies employed the state space-based approach as the GT mathematical model for the control system design. Only a few studies of the gain scheduling technique employing the thermodynamics-based model of a GT engine have been conducted. Gaudet [26] developed a thermodynamics-based dynamical model and proposed the gain scheduling control system of the GT engine considering engine performance limiters for marine applications. Yazar et al. [27] presented a mathematical model employing thermodynamics equations and developed the control system of the small turbojet aero engine. The transfer functions of operating conditions were computed using the data of fuel flow and speed. These data were collected from the simulation of the developed mathematical model running in the MATLAB environment. The proposed control system employed a gain scheduling technique to combine the adaptive neuro fuzzy inference system and modified PID. As compared to the state space-based motel, studies of the gain scheduling technique implemented to the thermodynamics-based model of the GT engine for the design purpose need to be studied further. The lack of the thermodynamic-model study is possibly due to the fact that the gain scheduling technique is a complex task that requires a computational strategy, as well as that the thermodynamics-based model of GT itself obviously requires a quite massive design workload [28].

Lin et al. [28] investigated the dynamics modeling and control design of a micro-GT. The control systems, which were a nonlinear and linear active disturbance rejection controller and a PID, were proposed. The nonlinear active disturbance rejection controller showed the best performance as compared to the other methods. Singh et al. [29] presented a thermodynamics-based mathematical model of a min SR-30 GT using state variable modeling. The modern control system was proposed to achieve the stability of the GT. The robust nonlinear fuel flow controller was developed using nonlinear dynamic inversion augmented with a dual extended Kalman filter. Sayedtabaii and Moradi [30] modified the fuzzy gas scheduling PID controller. The simplified Rowen dynamics model was applied as a GT dynamics model. Hybrid GA-PSO and classical fminsearch was implemented to tune the adaptive neuro-fuzzy interference system. The results demonstrated that the achievements of the proposed method were superior to the responses of some previously published methods.

According to the above literature review of GT control systems, some remarks regarding the development of the GT control system are: (1) The gain scheduling technique is a very promising approach to control the GT engine. (2) There are few studies of the gain scheduling technique applied to the thermodynamic model of GT engine. (3) Although the computation is more complex than that in the state space-based model, the thermodynamics model provides more flexibility and advantages for designing the small GT for achieving energy efficiency. (4) There are few studies of the design optimization of thermodynamics-based GT engine [31]. Therefore, this paper contributes to the development of computational techniques to achieve an optimal control system design of the thermodynamics-based model of the small GT engine. The gain-scheduling technique employing meta-heuristic optimizations is developed. Furthermore, the effect of using renewable fuel on the off-design performance and gain scheduling is observed.

Because the GT has a fuel boundary, the control system to manage the fuel rate within its safe operational area is essential for the GT engine. The optimization of the mini-GT engine control system, which is the gain scheduling technique of a PI controller with additional proportional gains, is proposed in this paper. The design of two-shaft GT engine for marine applications with 1.5 MW power output is studied. For performance limiters, the min-max strategies are applied to select the transient fuel flow rate. The thermodynamics-based model of the gas turbine engine is implemented to obtain the off-design performance and fuel graph boundary. The GT model and its control strategies are highly nonlinear containing a huge number of parameters. Using analytical solutions to optimize the GT may not be effective because of the computational time and local optimum issues. Optimization algorithms can be grouped into three categories, namely gradient-based algorithms, gradient-free algorithms, and artificial intelligence algorithms [32]. It is only the artificial intelligence algorithms, including the meta-heuristic optimization, free from the need of gradient information. Thus, using the meta-heuristic optimization for a gain tuning/scheduling controller is a very promising approach since these methods do not rely on the gradient of the function to obtain the optimal solution. This paper employs the meta-heuristic optimization technique, namely the WOA, to the designed control system of the two-shaft GT engine using the integral of time multiplied by absolute error (ITAE) performance index as the objective function.

The gain scheduling technique has been widely implemented to the GT engine; however, it has mainly been implemented to the state-space based model. The thermodynamics model, which is also known as the physics-based model, provides the detailed performance of the GT engine. The state-spaced based model may lose some essential behaviors of the GT engine [28]. To support the IMO goal of maritime clean energy, the methodology to design the new GT engine that has good performance in supporting the use of clean fuel is highly important. In the future, the new design of small/micro-GT engine that is optimal and adaptable to clean energy fuel will certainly play an important role. The thermodynamics-based model is necessary to design a new type of the GT engine because at the first step of the design, the data of the engine model is not yet available. This paper presents the computational strategy to optimize the gain scheduling control of the thermodynamics-based model of a small gas GT engine using meta-heuristic optimization.

The rest of the paper is organized as follows: Section 2 presents the dynamic modeling of the GT engine. The control system design is described in Section 3. Section 4 presents the meta-heuristic optimizations. The gain optimizations and detail objective functions are explained. Section 5 presents the results and discussions. The proposed gain scheduling is generated inside its fuel boundary. The performance of the meta-heuristic optimizations in optimizing the gain scheduling technique is observed. The effect of using bioethanol as the GT fuel on the GT thermodynamic performance and gain scheduling is also observed. The conclusions are presented in Section 6.

## 2. Dynamical Modeling

**Figure 2:**
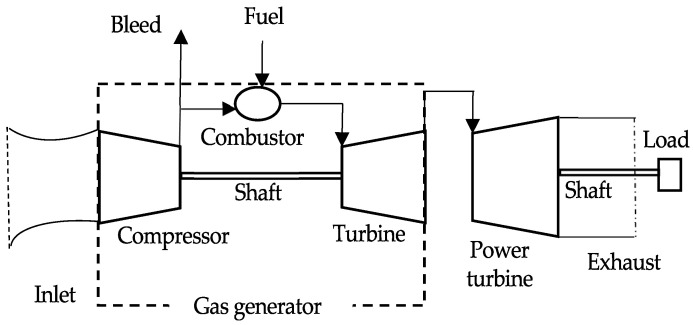
Two-shaft GT engine.

Figure 2 illustrates the two-shaft industrial GT engine studied in this paper. The components of the GT consist of the inlet, the compressor, the combustor, the turbine, the power turbine, the exhaust, the shaft, and the load.

### 2.1. Gas Turbine Dynamical Modeling

**Figure 3:**
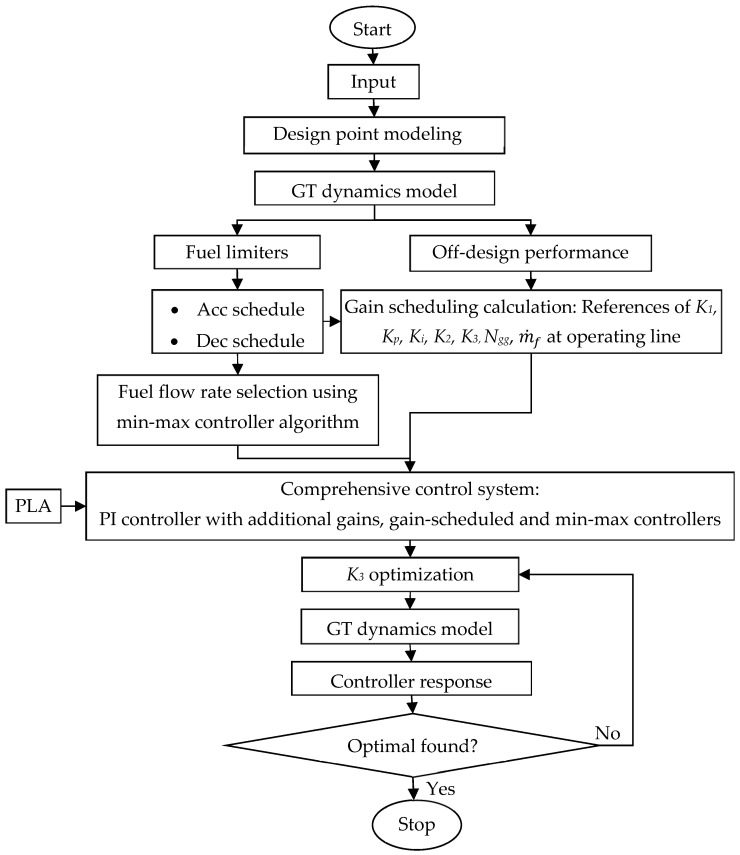
Flow chart of GT control system optimization.

This section presents the GT modeling, which is required to obtain the off-design performance and used to model the dynamics of the GT for the control system design. Figure 3 illustrates the step-by-step computation in designing the control system of a GT engine. Design point modeling is the initial step of this process to determine the designed operational parameter. The design point modeling can be obtained using the standard thermodynamics model of the GT components. The next step is the off-design performance modeling, which consists of the steady state and transient off-design performance. For this off-design performance, some iterative computations of compressor and turbine components are necessary. This section presents the modeling of the GT components, which requires the acquisition of the off-design performance, and which are used to model the dynamic of GT for control system design.

The loss of pressure in in the inlet component can be obtained as follows [33]:

(1) $$\Delta P_{inlet}={\left(\Delta P_{inlet}\right)}_{des}{\left(\frac{\left(\frac{{\dot{m}}_{a}\sqrt{T_{oa}R_{a}}}{P_{oa}}\right)}{{\left(\frac{{\dot{m}}_{a}\sqrt{T_{oa}R_{a}}}{P_{oa}}\right)}_{des}}\right)}^{2}$$

(2) $$P_{o1}=P_{oa}\left(1-\Delta P_{inlet}\right)$$

where $\Delta P_{inlet}$, ${\left(\Delta P_{inlet}\right)}_{des}$, ${\dot{m}}_{a}$, $T_{oa}$, $P_{oa}$, and *R_a_* are loss of pressure, loss of pressure design, inlet air mass flow, stagnation temperature at inlet intake, stagnation pressure at exit inlet, and gas constant for air, respectively.

#### 2.1.1. Compressor

**Figure 4:**
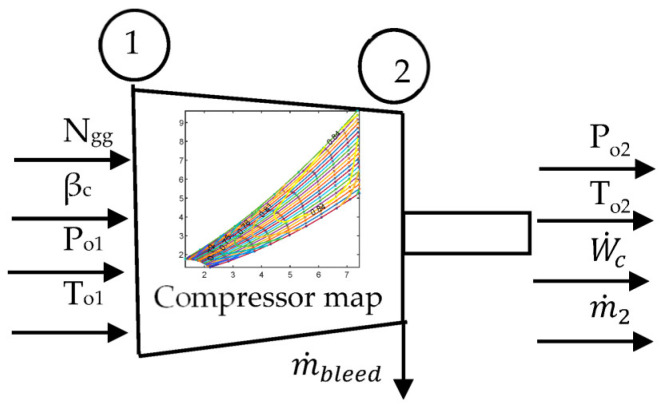
Compressor model.

The modeling of the compressor is illustrated in Figure 4. It is necessary to compute the compressor exit conditions. Given the spool speed and beta line, knowing the compressor inlet conditions, the exit conditions can be determined through the compressor map. At the initial stage of the GT design, the compressor is not available; however, it can be estimated using the published compressor map. In this study, a compressor map is estimated using the method proposed by Seller and Daniele [34].

The published compressor map is utilized, and using the design pressure ration, mass flow rate, and isentropic efficiency, the approximate compressor characteristics are determined as follows

(3) $$PR_{c}=\frac{PR_{c,des}-1}{PR_{c,map,des}-1}\left(PR_{c,map}-1\right)+1$$

(4) $${\left({\dot{m}}_{a}\sqrt{\theta /\delta }\right)}_{1}=\frac{{\left({\dot{m}}_{a}\sqrt{\theta /\delta }\right)}_{1,des}}{{\left({\dot{m}}_{a}\sqrt{\theta /\delta }\right)}_{map,des}}{\left({\dot{m}}_{a}\sqrt{\theta /\delta }\right)}_{map}$$

(5) $$\eta_{c,isen}=\frac{\eta_{c,isen,des}}{\eta_{c,isen,map,des}}\left(\eta_{c,isen,map}\right)$$

where $PR_{c}$, $PR_{c,des}$, $PR_{c,map}$, and $\eta_{c,isen}$ are the compressor pressure ratio, design of compressor pressure ratio, compressor pressure ratio of the map, and isentropic efficiency of the compressor, respectively.

The auxiliary coordinates, namely the beta lines, $\beta_{c}$, are added to the compressor map. Then, the compressor characteristics are computed using following equations:

(6) $$PR_{c,map}=fn\left(\beta_{c},\;\%N_{c}\right)$$

(7) $${\left({\dot{m}}_{a}\sqrt{\theta /\delta }\right)}_{map}=fn\left(\beta_{c},\;\%N_{c}\right)$$

(8) $$\eta_{c,isen,map}=fn\left(\beta_{c},\;\%N_{c}\right)$$

where:

$$\theta =T_{o}/288.15\;K\;\delta =P_{o}/101.325\;kPa\;\%N_{c}=\frac{N_{gg}∕\sqrt{\theta }}{{\left(N_{gg}∕\sqrt{\theta }\right)}_{des}}$$

Exit temperature of the compressor can be computed using the definition of the isentropic efficiency, as follows

(9) $$\eta_{c,isen}=\frac{h\left(T_{o2s}\right)-h\left(T_{o1}\right)}{h\left(T_{o2}\right)-h\left(T_{o1}\right)}$$

where *h*, *T_o_*_2*s*_, and *T_o_*_2_ are specific enthalpy of air, ideal compressor exit temperature, and compressor exit temperature, respectively.

The ideal exit temperature, *T_o_*_2*s*_, is necessary for solving the above equation. The standard specific entropy can be used as follows

(10) $$S_{std}\left(T_{o2s}\right)-S_{std}\left(T_{o1}\right)=R_{a}ln\left(PR_{c}\right)$$

(11) $$S_{std}\left(T\right)=\Psi \left(T\right)\left(\frac{R_{a}}{log\left(e\right)}\right)$$

where $S_{std}$ and Ψ are standard specific entropy and specific entropy of air, respectively.

The value of *T_o_*_2*s*_ can be obtained by solving Equations (10) and (11) iteratively. After *T_o_*_2*s*_ is found, the value of the compressor exit temperature, *T_o_*_2_, can be obtained by solving Equation (9) iteratively. This paper uses the model used in [33] for the computation of $S_{std}\left(T\right)$, $\Psi \left(T\right)$, *C_p_*(*T*), and *h*(*T*).

#### 2.1.2. Combustor

**Figure 5:**
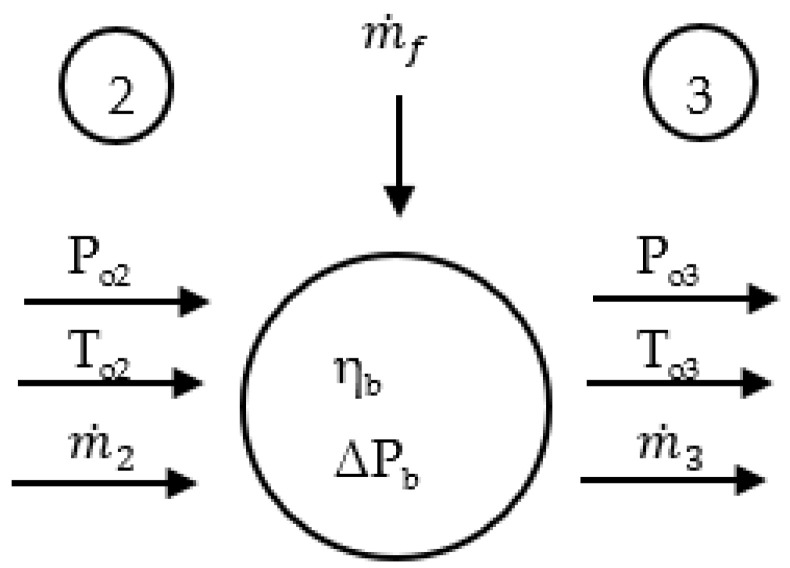
Combustor model.

Figure 5 illustrates the combustor modeling. To calculate *T_o_*_3_, the following equation, which is derived from the definition of the combustion efficiency, is considered

(12) $$h\left(T_{o3}\right)=\frac{{\dot{m}}_{f}.HV.\eta_{b}+\left({\dot{m}}_{2}\right)h\left(T_{o2}\right)}{\left({\dot{m}}_{2}+{\dot{m}}_{f}\right)}$$

where ${\dot{m}}_{f}$, HV, and $\eta_{b}$ are the fuel flow, fuel heating value, and combustion efficiency, respectively.

This paper approximates the combustion efficiency using the Walsh and Fletcher model [33].

The combustor exit pressure can be determined as follows:

(13) $$\Delta P_{b}={\left(\Delta P_{b}\right)}_{des}{\left(\frac{\left(\frac{{\dot{m}}_{2}\sqrt{T_{o2}R_{a}}}{P_{o2}}\right)}{{\left(\frac{{\dot{m}}_{2}\sqrt{T_{o2}R_{a}}}{P_{o2}}\right)}_{des}}\right)}^{2}$$

(14) $$P_{o3}=P_{o2}\left(1-\Delta P_{b}\right)\;$$

#### 2.1.3. Gas Generator Turbine

**Figure 6:**
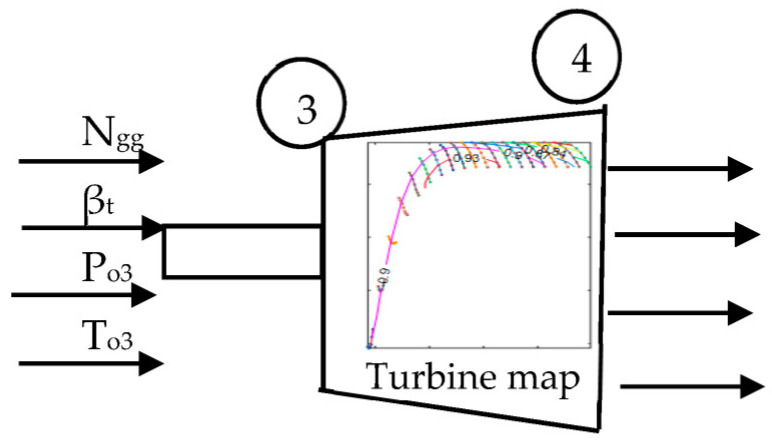
Gas generator turbine model.

As for the compressor, the turbine map is also very important for the turbine dynamic model. Since the turbine map is not available at the initial stage of GT design, the estimation of the turbine characteristic is necessary. Figure 6 shows the gas generator turbine modeling.

Using the published turbine map, the method developed by Seller and Daniele [34] is employed:

(15) $$PR_{t}=\frac{PR_{t,des}-1}{PR_{t,map,des}-1}\left(PR_{t,map}-1\right)+1$$

(16) $${\left(\dot{m}\sqrt{\theta /\delta }\right)}_{3}=\frac{{\left(\dot{m}\sqrt{\theta /\delta }\right)}_{3,des}}{{\left(\dot{m}\sqrt{\theta /\delta }\right)}_{t,map,des}}{\left(\dot{m}\sqrt{\theta /\delta }\right)}_{t,map}$$

(17) $$\eta_{t,isen}=\frac{\eta_{t,isen,des}}{\eta_{t,isen,map,des}}\left(\eta_{t,isen,\;map}\right)$$

The following equations need to be generated:

(18) $$PR_{t}=fn\left(\beta_{t},\;\%N_{t}\right)$$

(19) $${\left(\dot{m}\sqrt{\theta /\delta }\right)}_{3}=fn\left(\beta_{t},\;\%N_{t}\right)$$

(20) $$\eta_{t,isen}=fn\left(\beta_{t},\;\%N_{t}\right)$$

$$\%N_{t}=\frac{N_{gg}/\sqrt{\theta }}{{\left(N_{gg}/\sqrt{\theta }\right)}_{des}}$$

where $PR_{t}$, $PR_{t,des}$, $PR_{t,map}$, $\eta_{t,isen}$, and $\beta_{t}$ are the gas generator turbine pressure ratio, the design of gas generator turbine pressure ratio, the gas generator turbine pressure ratio of the map, and the isentropic efficiency of gas generator turbine, respectively.

To obtain the exit gas generator (GG) turbine temperature, the ideal gas generator turbine temperature is determined using the standard entropy equation, as follows:

(21) $$S_{std}\left(T_{o4s}\right)-S_{std}\left(T_{o3}\right)=R_{a}ln\left(\frac{1}{PR_{t}}\right)$$

(22) $$S_{std}\left(T\right)=\Psi \left(T\right)\left(\frac{R_{a}}{log\left(e\right)}\right)$$

After the $T_{o4s}$ value has been obtained, the value of the gas generator turbine exit temperature can be computed iteratively using the enthalpy function, as follows:

(23) $$h\left(T_{o4}\right)=h\left(T_{o3}\right)-\eta_{t,isen}\left(h\left(T_{o3}\right)-h\left(T_{o4s}\right)\right)$$

where $\eta_{t,isen}$ is the isentropic efficiency of gas generator turbine.

The GG turbine does not have blade cooling, so the turbine discharge mass flow is as follows:

(24) $${\dot{m}}_{4}={\dot{m}}_{3}$$

The GG exit pressure can be obtained using following equation:

(25) $$P_{o4}=\frac{P_{o3}}{PR_{t}}$$

#### 2.1.4. Power Turbine

To obtain the power turbine characteristics, the same procedures used for the GG turbine computations are applied to the power turbine.

For the exhaust component, the pressure can be expressed as follows:

(26) $$P_{o6}=P_{o5}\left(1-\Delta P_{exhaust}\right)$$

The ducting pressure loss equation given by Kurzke [35] is employed as follows:

(27) $$\Delta P_{exhaust}={\left(\Delta P_{exhaust}\right)}_{des}{\left(\frac{\left(\frac{{\dot{m}}_{5}\sqrt{T_{o5}R_{5}}}{P_{o5}}\right)}{{\left(\frac{{\dot{m}}_{5}\sqrt{T_{o5}R_{5}}}{P_{o5}}\right)}_{des}}\right)}^{2}$$

### 2.2. Off-Design Performance

To obtain the off-design performance of the GT engine, each component of the GT engine should be linked together to assemble an engine.

#### 2.2.1. Steady State Performance

Off-design steady state performances at operating conditions are necessary to compute the gains reference in the gain scheduling technique. An iterative computation is necessary to ensure the matching between each component. This paper employs the Newton–Raphson method as the iterative method with an error tolerance of $1\times 10^{-8}$, using sum of square error as an error metric. There are five input iteration variables, namely *β_c_*, TIT, *β_t_*, *β_pt_*, *β_pt_*, and %*N_pt_*. Five iteration errors considering the compatibility flow and compatibility work need to be solved. These iteration errors are the compatibility of the flow between the combustor and gas generator turbine, the compatibility of the work between the compressor and the gas generator turbine, the compatibility of the flow between the gas generator turbine and the power turbine, the compatibility of the work between the turbine and the propeller, and the compatibility of the pressure between the exhaust and the ambient condition.

#### 2.2.2. Transient Performance

The transient performance deals with the changing of the operating conditions needed to accelerate or decelerate from one steady-state operation condition to another steady-state operating point. Differing from the steady-state performance, the mismatch is necessary to permit the engine to accelerate or decelerate. For a two-shaft GT engine, this mismatch can be obtained between the compressor and gas generator turbine or between the power turbine and the load.

Based on the change of the speed and pressure for a given time interval Δ*t*, the prediction of the new operating conditions can be expressed as follows:

(28) $$N_{gg,new}=N_{gg,old}+\frac{dN_{gg}}{dt}\Delta t\;N_{pt,new}=N_{pt,old}+\frac{dN_{pt}}{dt}\Delta t\;P_{o2,new}=P_{o2,old}+\frac{dP_{o2}}{dt}\Delta t$$

where $\frac{dN_{gg}}{dt}$, $\frac{dN_{pt}}{dt}$, and $\frac{dP_{o2}}{dt}$ are the rate of change of the gas generator spool speed, power turbine spool speed, and *P_o_*_2_, respectively.

In this paper, the inter-component volume method developed by Fawke and Saravanamuttoo [36] is employed in the transient computation using the Newton–Raphson method for computing the controller response.

### 2.3. Model Validation

To validate the GT dynamics model, existing commercial GTs are selected to compare the results of their technical data and the model output. The fuel used in validation is marine diesel oil, as shown in [37]. The first GT which is used to validate the GT dynamics model is LM2500. It is a two-shaft GT engine manufactured by General Electric (Boston, MA, USA). Table 1 shows the technical data of the LM2500. Table 2 presents the results of the GT model output. The second GT which is used to validate the GT dynamics model is SGT-100. It is a two-shaft GT engine manufactured by Siemens (Munich, Germany). Table 3 shows the technical data of the SGT-100. Table 4 presents the result of the GT model output. The errors are within the range of 5.7% and 3.28% for the LM2500 and the SGT-100, respectively. Thus, both of these error values are acceptable.

## 3. Control System Design

Since the type of GT is two-shaft, the requirement of the control system is to control the turbine speed through fuel flow. Gain scheduling techniques apply the linearization principle. The interpolation is employed between a set of linear controllers. The gains as scheduling parameters are arranged as function of scheduling variables or plant variables.

### 3.1. Gain Scheduling Technique of a PI Controller with Additional Proportional Gains

**Figure 7:**
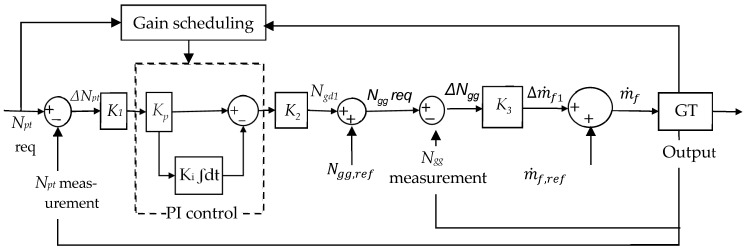
PI controller with gain scheduling.

Figure 7 illustrates the gain scheduling technique of a PI controller, with additional gain applied to the GT engine.

$K_{p}$ and $K_{i}$ gains are defined from the second-order system as follows [26]:

(29) $$K_{p}=2\xi \omega_{n}\;\;\;\;;\;K_{i}=\omega_{n}{}^{2}\tau$$

where τ is time constant, which is the time to achieve a 63.2% demand change.

The damping ratio, ξ, and natural frequency, $\omega_{n}$, are obtained as follows:

(30) $$M_{p}=e^{\left(\xi /\sqrt{\left(1-\xi^{2}\right)}\right)\pi }$$

(31) $$t_{set}=\frac{4}{\xi \omega_{n}}\;\;\;\;\left(2\%\;\mathrm{criterion}\right)\;\;\;\;\;\;\mathrm{or}\;t_{set}=\frac{3}{\xi \omega_{n}}\;\;\;\;\left(5\%\;\mathrm{criterion}\right)$$

where $M_{p}$ and *t_set_* are the maximum overshoot and settling time, respectively.

Besides *K_p_* and *K_i_*, there are additional proportional gains, namely $K_{1}$, $K_{2}$, and $K_{3}$. These gains are modeled as the function of the following parameter:

(32) $$K_{1}=\frac{1}{\Delta N_{pt}}\;\;$$

(33) $$K_{2}=\Delta N_{gg}\;\;\;$$

(34) $$\;K_{3}=a\frac{\Delta {\dot{m}}_{f}}{\Delta N_{gg}}$$

where

$$\Delta N_{pt}=N_{pt,max}-N_{pt,min}\;\Delta N_{gg}=N_{gg,max}-N_{gg,min}\;\Delta {\dot{m}}_{f}={\dot{m}}_{f,max}-{\dot{m}}_{f,min}$$

The gains Equations (29) and (32)–(34) need to be computed at operating lines to obtain the reference of the acceleration and deceleration gain schedules. The global controller gains are computed through interpolation of these reference gain schedules.

(35) $$\mathrm{References}\;\mathrm{of}\;\mathrm{gains}\;K_{\mathrm{ref}}\;\mathrm{are}\;\mathrm{computed}\;\mathrm{as}\;\mathrm{step}\;\mathrm{response}\;\mathrm{analysis}:\;K_{ref}=\left[K_{p,ref}\;\;\;K_{i,ref}\;\;\;K_{1,ref}\;\;\;K_{2,ref}\;\;\;K_{3,ref}\right]$$

where *K_ref_* is the references of gains *K*.

Gains *K*, $K=\left[K_{p}\;K_{i}\;K_{1}\;K_{2}\;K_{3}\right]$, are calculated as the interpolation of *K_ref_* as the function of engine spool speed:

(36) $$K=interp\left(K,\Delta N_{gg},K_{ref},\Delta N_{gg,ref}\right)\;\;$$

The acceleration and deceleration fuel schedules determine the maximum and minimum total fuel flow allowed for safe transient operations. This paper employs the acceleration and deceleration schedules as obtained in [26].

### 3.2. Min-Max Controller

The demand for GG spool speed is defined as follows:

(37) $$N_{gd}=min\;\left[N_{gd1}\;\;\;N_{gd2}\;\;N_{gd3}\;\;N_{gd4}\right]$$

where *N_gd_*_1_, *N_gd_*_2_, *N_gd_*_3_, and *N_gd_*_3_ are the GG spool speed from the PI control, which linearly modifies the *N_gd_*_1_ between 100% and 0% based on temperature protection, GG overspeed protection, and maximum PT overspeed protection, respectively.

The fuel flow demand when the engine is accelerated is selected as the lowest fuel flow, as follows:

(38) $${\dot{m}}_{f,demand/acc}=min\;\left[\begin{array}{cc} \Delta {\dot{m}}_{f}+{\dot{m}}_{f,ref} & acc_{sched} \end{array}\right]$$

$$\Delta {\dot{m}}_{f}=\min\;\left[\Delta {\dot{m}}_{f1}\;\;\;\Delta {\dot{m}}_{f2}\;\;\Delta {\dot{m}}_{f3}\right]$$

where $\Delta {\dot{m}}_{f1}$ is the fuel flow signal from the PI controller. $\Delta {\dot{m}}_{f2}$ and $\Delta {\dot{m}}_{f3}$ linearly modify the $\Delta {\dot{m}}_{f1}$ between 100% and 0%, based on minimum speed and flameout protections, respectively. $acc_{sched}$ is the acceleration schedule.

The fuel flow demand when the engine is decelerated is select as highest fuel flow, as follows:

(39) $${\dot{m}}_{f,demand/dec}=max\;\left[\begin{array}{cc} {\dot{m}}_{f,demand/acc} & dec_{sched} \end{array}\;\;\right]$$

where $acc_{sched}$ is the deceleration schedule.

Equations (37)–(39) represent the protective control to avoid engine damage.

## 4. Meta-Heuristic Optimization

The ITAE performance index is used as the objective function. The ITAE performance index can be expressed as follows

(40) $$ITAE=\int_{0}^{T}t\left|e\left(t\right)\right|dt$$

This paper considers the error $e\left(t\right)$ before it is inputted into the PI controller as follows:

(41) $$e\left(t\right)=K_{1}\left(N_{pt,req}-N_{pt}\right)$$

where *N_pt,req_* and *N_pt_* are the requirements of the power turbine spool speed and the power turbine spool speed, respectively.

### 4.1. Gain Optimization

The proposed comprehensive control system can be expressed as the constraint optimization problem where the optimization objective is to minimize the ITAE as follows:

$$min\;\int_{0}^{T}t\left|e\left(t\right)\right|dt$$

Subject to:

0.1<a<24

where

$$K_{3}=a\frac{\Delta {\dot{m}}_{f}}{\Delta N_{gg}};\;K_{1}=\frac{1}{\Delta N_{pt}};\;K_{2}=\Delta N_{gg}$$

$$K_{p}=2\xi \omega_{n};\;K_{i}=\omega_{n}{}^{2}\tau$$

$$e\left(t\right)=K_{1}\left(N_{pt,req}-N_{pt}\right)$$

(42) $$N_{gd}=min\;\left[N_{gd1}\;\;\;N_{gd2}\;\;N_{gd3}\;\;N_{gd4}\right]$$

(43) $$\Delta {\dot{m}}_{f}=min\;\left[\Delta {\dot{m}}_{f1}\;\;\;\Delta {\dot{m}}_{f2}\;\;\Delta {\dot{m}}_{f3}\right]$$

(44) $${\dot{m}}_{f}=max\;\left[{\dot{m}}_{f,\;demand\;\;}\;dec_{sched}\;\right]$$

$${\dot{m}}_{f,\;demand}=min\left[\left(\Delta {\dot{m}}_{f}+{\dot{m}}_{f,ref}\right)\;\;\;acc_{sched}\right]$$

$$K_{ref}={\left[K_{p,ref}\;\;\;K_{i,ref}\;\;\;K_{1,ref}\;\;\;K_{2,ref}\;\;\;K_{3,ref}\right]}^{T}$$

$$K=\left[K_{p}\;\;\;K_{i}\;\;\;K_{1}\;\;\;K_{2}\;\;\;K_{3}\right]$$

$$K=interp\left(K,\Delta N_{gg},K_{ref},\Delta N_{gg,ref}\right)$$

where *K_ref_* is the references of gains *K*.

The references of gains *K_ref_* are computed as a step response analysis and the gains *K* are calculated as the interpolation of *K_ref_* as a function of the engine spool speed. Equations (42)–(44) represent the protective control to avoid engine damage. *N_gd_*_1_ is the signal of the GG spool speed from the PI control. *N_gd_*_2_, *N_gd_*_3_, and *N_gd_*_3_ linearly modify the *N_gd_*_1_ between 100% and 0%, based on temperature protection, GG overspeed protection, and maximum PT overspeed protection, respectively. $\Delta {\dot{m}}_{f1}$ is the fuel flow signal from the PI controller. $\Delta {\dot{m}}_{f2}$ and $\Delta {\dot{m}}_{f3}$ linearly modify the $\Delta {\dot{m}}_{f1}$ between 100% and 0%, based on the minimum speed and flameout protections, respectively. $acc_{sched}$ and $dec_{sched}$ are the acceleration schedule and deceleration schedule, respectively. Acceleration and deceleration schedules are obtained during off-design performance computation. Acceleration and deceleration fuel schedules determine the maximum and minimum total fuel flow permitted during transient operations.

### 4.2. Genetic Algorithm

There are three main operators in the GA: reproduction, crossover, and mutation. The optimization parameter is converted to the chromosomes and coded in the evolution process. Using the real code GA, the chromosome is represented as the number in sequence. The selection is the process to choose two individuals in the population as parents for mating to form the new offspring. A crossover is a process of randomly picking one or more individuals as parents and swapping the segments of the parents. This paper uses random resetting mutation. In this scheme, a randomly chosen gene is assigned to be exchanged with a random value. A detailed description of the procedure regarding the GA can be referenced in [39].

### 4.3. Whale Optimization Algorithm

The WOA is the meta-heuristic optimization developed by Mirjalili et al. [40] in 2015. It is inspired by the bubble-net hunting strategy of humpback whales.

Humpback whales can recognize the location of prey and then encircle them. The current candidate for the best solution is presumed as the target prey. The search agents will update their positions toward the best search agent. Humpback whales search for prey randomly based on their positions in regards to each other. In the exploitation phase, the position of the search agent is updated according to a randomly chosen search agent instead of the current best search agent. A detailed description of the procedure of the WOA can be referenced in [40].

According to the design control system presented in the previous sections, the parameter *a* in Equation (34) is the chromosome in the GA and the search agent in the WOA. Following the procedure of the GA and WOA, the fitness value is calculated and evaluated as Equation (41).

## 5. Results and Discussion

This section presents the results of the developed control strategy in designing a 1.5 MW two-shaft GT engine. The off-design steady state and transient performances are presented first. The control system performance for acceleration case and deceleration case are investigated. The comparison when the scheduled gains are constant is observed. Finally, the performance of the off-design and control system when the biofuel is used is observed.

### 5.1. Fuel Flow Boundary

**Figure 8:**
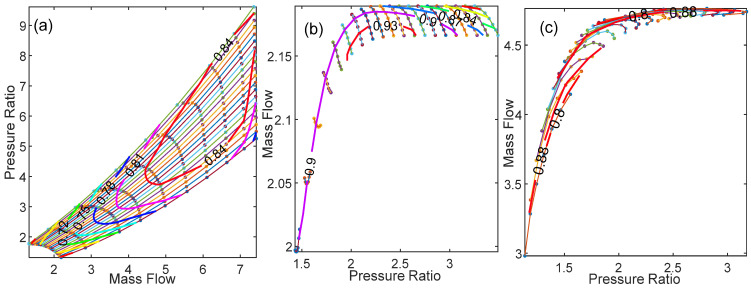
(**a**) Compressor map; (**b**) turbine map; (**c**) power turbine map.

**Figure 9:**
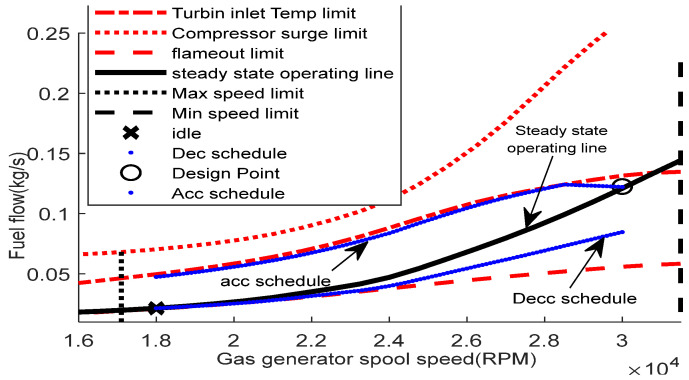
Fuel boundary graph.

The inputs of the GT dynamic model are presented in Table 5. The values of inputs are obtained from the design point performance adaptation, as in [41]. Figure 8 shows the results of the compressor map, the gas generator turbine map, and the power turbine map using the Sellers and Daniele method [34]. Using the GT dynamical model presented in Section 2, Figure 9 shows the fuel flow boundary of the designed 1.5 MW GT engine. The fuel flow boundary is obtained by computing the steady state performance of various conditions, which are normal condition, maximum TIT, maximum spool speed, minimum spool speed, minimum flame out limit, and compressor surge, plotting them in the same graph. The acceleration fuel schedule based on the maximum fuel schedule within the acceleration limit is selected, while for the deceleration schedule, a 30% under-fueling margin is chosen.

For the controller response, the control system is considered to continue the control demand until the following condition is not satisfied

(45) $$\left(\left|\frac{dN}{dt}\right|\leq 1+\left|\frac{dN_{pt}}{dt}\right|\leq 1\right)<2$$

where $\frac{dN}{dt}$ and $\frac{dN_{pt}}{dt}$ are the rate of change of GG turbine speed and the rate of change of power turbine speed, respectively.

The condition in Equation (45) is the logical expression of the control demand of the GG turbine and the power turbine. In this case, the condition of the rate of change of the GG turbine speed: $\left|\frac{dN}{dt}\right|=1$, and the rate of change of the power turbine speed: $\left|\frac{dN}{dt}\right|=1$, represent a fulfillment condition to indicate that there is no more request for fuel flow change.

### 5.2. Control System

All computations of the GT dynamics model and the GT control system optimization are performed by writing the computer code in the MATLAB environment. The GA and WOA use six individuals in the population. For the GA, this paper uses a selection rate of 0.5 and a mutation rate of 0.08. The optimization parameter, *a*, is searched within the search area [0.1, 24].

#### 5.2.1. Case 1: Acceleration PLA = 25%

**Figure 10:**
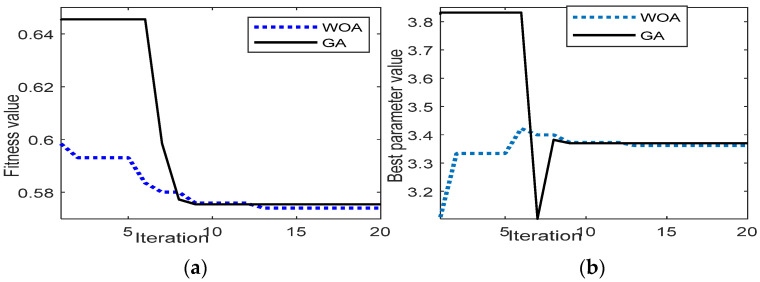
(**a**) Fitness value; (**b**) best parameter value evolution.

A power lever angle (PLA) is used as the input to the control system. The PLA is defined as the percentage of the total power turbine spool speed. The control system optimization of the GT acceleration from the idle speed to 0.25 of PLA is observed in this section. Using 20 iterations, Figure 10a shows the fitness value obtained by GA and WOA. It can be observed that the WOA shows better results than those of the GA. Details of the best fitness value and the best parameter value are presented in Table 6. Figure 10b shows the evolution of the best parameter value at each iteration.

**Figure 11:**
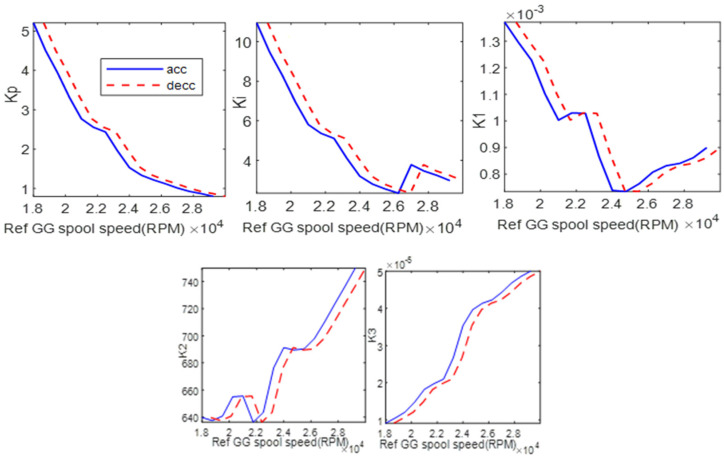
Gains scheduling references, *a* = 3.3594.

Figure 11 shows the gains scheduling using the value of *a* = 3.3594. The gains are computed using 16 local linear controllers of acceleration and deceleration gains. These gains scheduling data are used to interpolate the global gains values, with PLA as the input to the control system.

**Figure 12:**
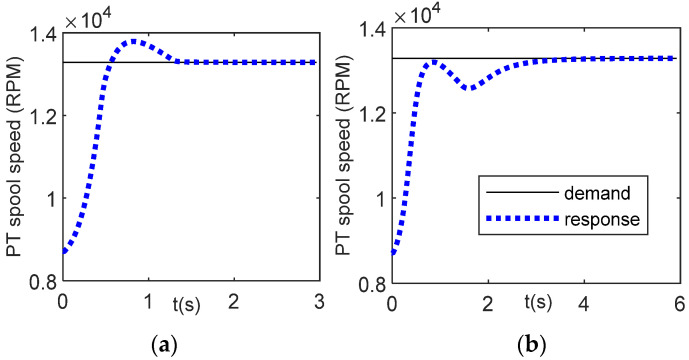
Controller response, PLA = 0.25; (**a**) optimal parameter *a* = 3.3594; (**b**) unoptimized parameter *a* = 1.

Figure 12a,b shows the controller response for PLA = 25% using values of *a* = 3.3594 and *a* = 1, respectively. The value of *a* = 1 is the unoptimized gain scheduling. Using this value, if the fitness value, Equation (41), is computed, the result of fitness value is 2.0589, which is quite large as compared with the fitness value of the optimal *a*, *a* = 3.3594, i.e., 0.5737 (Table 2). From controller response graphs, it can be observed that for *a* = 1, the time to condition (43) to stop is longer than that of the optimal parameter condition. It requires only 2.96 s for *a* = 3.3594 while for *a* = 1, the time to complete it is 5.92 s.

#### 5.2.2. Case 2: Deceleration with PLA = [0.5 0]

**Figure 13:**
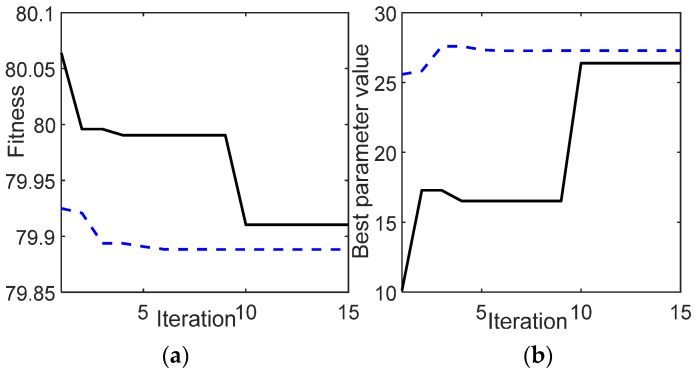
Case 2, deceleration from maximum speed to idle speed, PLA = [0.5 0]; (**a**) fitness value; (**b**) best parameter value evolution.

The second study case is the deceleration from the maximum power turbine speed until reaching the idle speed with PLA = [0.5 0]. Figure 13a shows the fitness value obtained by the GA and WOA. As in Case 1, the WOA shows better performance than that of the GA, whereas during 15 iterations, the WOA shows the minimum fitness value, as compared to the GA result. Figure 13b illustrates the best parameter value reached at each iteration. The detail of the best parameter and fitness value is presented in Table 7.

**Figure 14:**
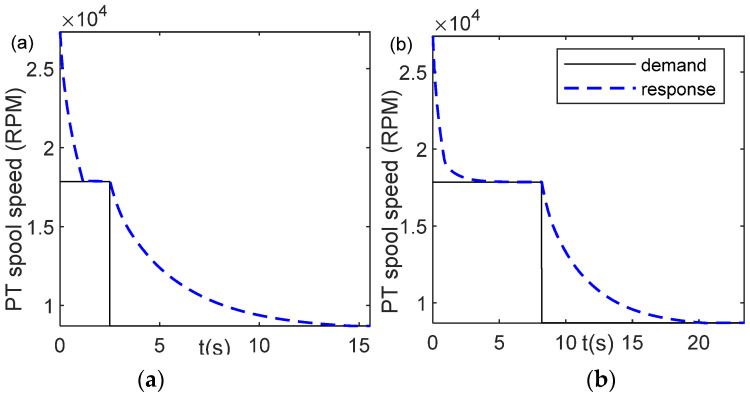
Controller response for Case 2, deceleration from maximum speed to idle speed, PLA = [0.5 0]; (**a**) optimal parameter *a* = 27.2848; (**b**) unoptimized parameter, *a* = 1.

Figure 14a shows the power turbine response using the optimal parameter *a* = 27.2848. The condition of Equation (44) is a stop at *t* = 15 s. To confirm that the WOA has succeeded in optimizing the previous work, the controller response using the original value of *a* = 1 is investigated, as shown in Figure 14b. Using this value, the stopping condition of the fuel demand is achieved at *t* = 23.41 s. Thus, applying the meta-heuristic optimization, namely the WOA, the time to achieve the target PLA is faster than without the optimization scheme.

#### 5.2.3. Specific Fuel Consumption during Acceleration and Deceleration Modes

**Figure 15:**
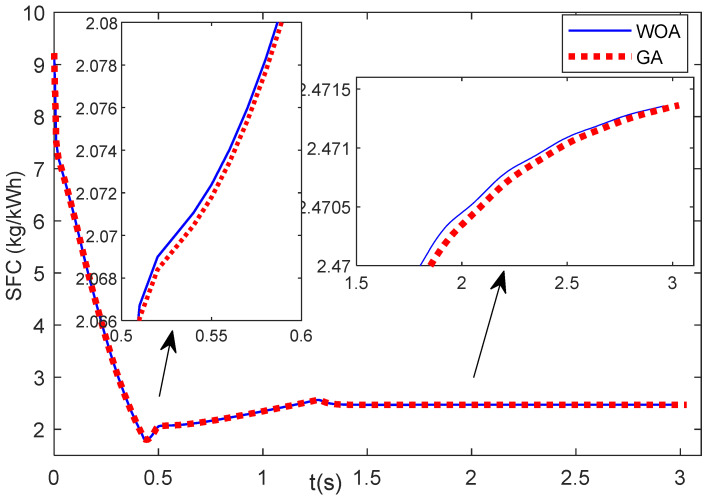
SFC of Case 1.

**Figure 16:**
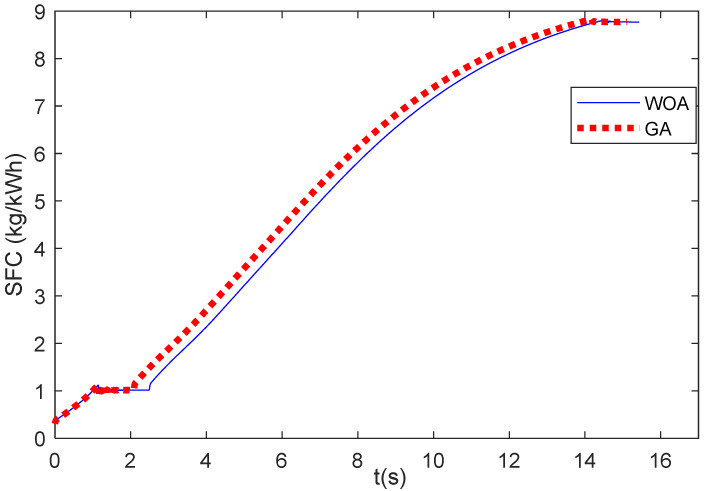
SFC of Case 2.

This section observes the specific fuel consumption (SFC) during the engine acceleration and deceleration phases. Figure 15 shows the SFC during the engine acceleration of Case 1. It shows that the SFC obtained from the results of GA and WOA does not show much difference. The SFC values of the WOA are a little above the SFC values of the GA. Figure 16 illustrates the SFC values during the engine deceleration of Case 2. It shows that the SFC value of the GA results is higher than that of the WOA results.

This research uses the ITAE as the objective function and does not consider the SFC in the objective function. For the SFC of Case 1, the WOA results are a little higher the GA results. However, it is only a very small difference. For Case 2, the SFC values of the WOA are lower than those of the GA. It seems that the SFC results cannot be predicted from the results of the ITAE objective function. Thus, for future research, to obtain the minimum SFC during the acceleration/deceleration mode, the SFC can be considered in the objective function so that it becomes the multi-objective function optimization problem. Another possibility to minimize the SFC is to consider the optimization of the acceleration and deceleration schedules.

### 5.3. Comparison with Constant Gains

**Figure 17:**
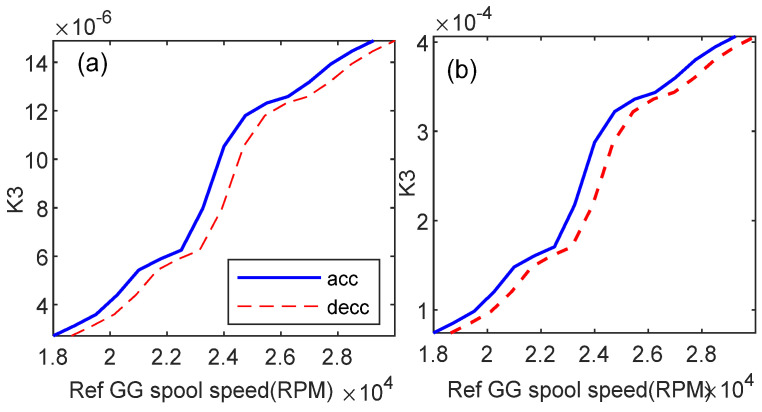
*K*_3_ (**a**) optimal parameter *a* = 3.3594; (**b**) unoptimized parameter *a* = 1.

With the scheme of Equation (34), different values of parameter *a* correlate with different *K*_3_ scheduling. Other gains have same results of scheduling graphs as in Figure 11. Figure 17 shows the *K*_3_ scheduling for *a* = 3.3594 and *a* = 1. The PI controller can be divided into two groups of gain tuning: constant gain and scheduled gain. It is generally known that tuning the gain controller is a difficult task.

**Figure 18:**
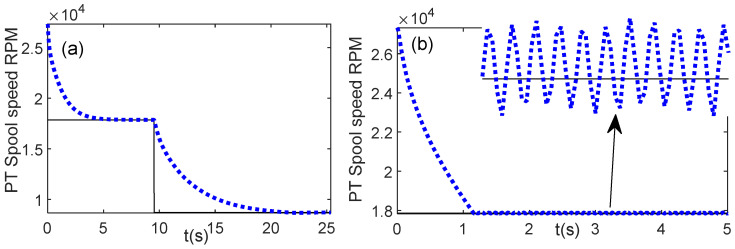
Controller response (**a**) [*K_p_ K_i_ K*_1_ *K*_2_ *K*_3_] = [2 4.2 0.0008 720 0.000004]; (**b**) [*K_p_ K_i_ K*_1_ *K*_2_ *K*_3_] = [3 6.3 0.001 650 0.0002].

Gain scheduling reference graphs can be used to investigate the appropriate gains values which result in a stable response system. To investigate this behavior, constant gains tuning is selected from Figure 11 and Figure 17. Figure 18a illustrates the controller response using the constant gains [*K_p_ K_i_ K*_1_ *K*_2_ *K*_3_] = [2 4.2 0.0008 720 0.000004] for Case 2. It shows that the system is stable, when time needed to achieve the target PLA is 25.35 s. Figure 18b illustrates the controller response using the constant gains [*K_p_ K_i_ K*_1_ *K*_2_ *K*_3_] = [3 6.3 0.001 650 0.0002] for Case 2. It shows that the system exhibits an oscillation with constant amplitude in the power turbine speed spool response. This kind of system response is not expected, since the controlled variable will not reach a stable condition. Both gains have been selected within the area of gain scheduling, but the results do not always exhibit the desired stability.

### 5.4. Biofuel

The previous section has shown that meta-heuristic optimizations have succeeded in optimizing the gain scheduling of the thermodynamics-based model of the GT engine when diesel No. 1 is used as the fuel. According to standard ASTM D 975, diesel No. 1 fuel fulfills the requirement for SOx emission, i.e., less than 5000 ppm [42]. It has been suggested that to support clean and sustainable marine transportation, the gas turbine should be designed to have at least dual fuel capability, especially supporting the renewable fuel system [43,44]. Multiple fuel capability, i.e., particularly for the biofuel, requires the modification of the GT element regarding the storage and delivery of the fuel system [9]. Therefore, the GT control system, which correlates with the fuel flow requirement during engine operation, is an important system to achieve the success toward the renewable energy system for the GT engine. This section investigates the performance of the GT engine when it uses biofuel, i.e., bioethanol, as the fuel. Bioethanol is a colorless, biodegradable, highly flammable, and low toxicity liquid. Using the bioethanol as the GT fuel has some advantages, not only because of the low emission level and a sulfur-free characteristics [3,45,46], as compare with the conventional fuel, but also because it is easier to transport and store [47].

#### 5.4.1. Fuel Flow Boundary

The computation of the thermodynamics-based dynamic model is conducted when the GT input parameters are still the same as those for the diesel No. 1 fuel, except for the fuel heating value. The change in the fuel exerts an effect because the lower heating value is reduced to 27,200 kJ/kg for the bioethanol. Table 8 presents the design point calculation comparison between diesel No. 1 and bioethanol. It shows that using the same GT parameters input, the shaft power obtained from the bioethanol is higher than that of the diesel No. 1; however, the SFC is also increased significantly. Fuel flow is also increased, while the thermal efficiency does not show much difference.

**Figure 19:**
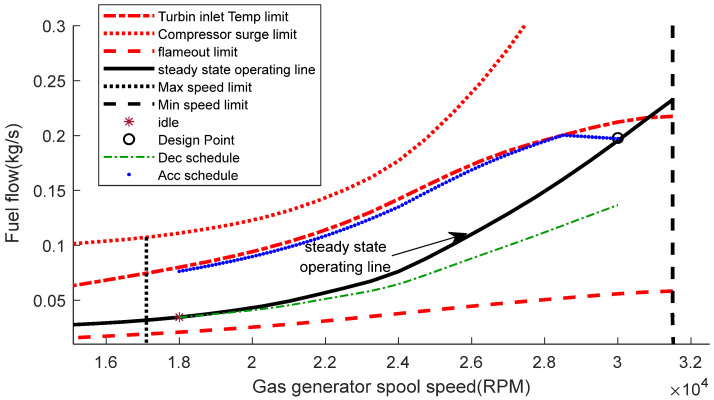
Fuel boundary graph using bioethanol as fuel.

**Figure 20:**
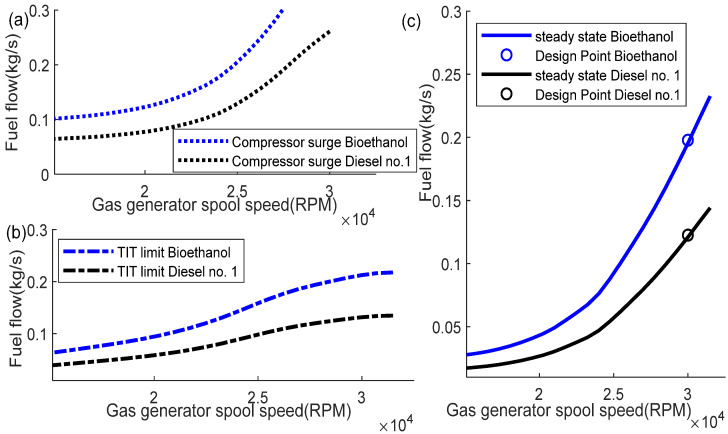
Comparison of diesel No. 1 and bioethanol; (**a**) compressor surge limit; (**b**) TIT limit; (**c**) steady state operating line.

Figure 19 shows the fuel boundary of the designed GT engine using bioethanol as the fuel. It shows that the lines of the compressor surge limit, the TIT limit, and the steady state operating line have been increased. Details of the comparison of the compressor surge limit, the TIT limit, and the steady state operating line are shown in Figure 20a–c.

#### 5.4.2. Effect to Gain Scheduling

**Figure 21:**
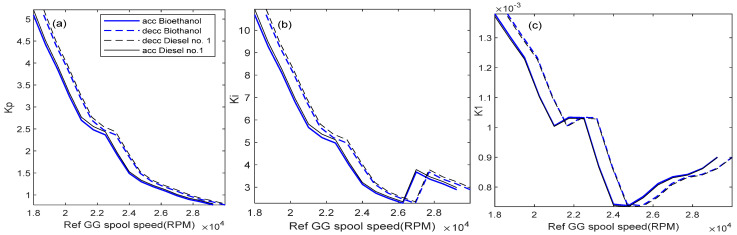

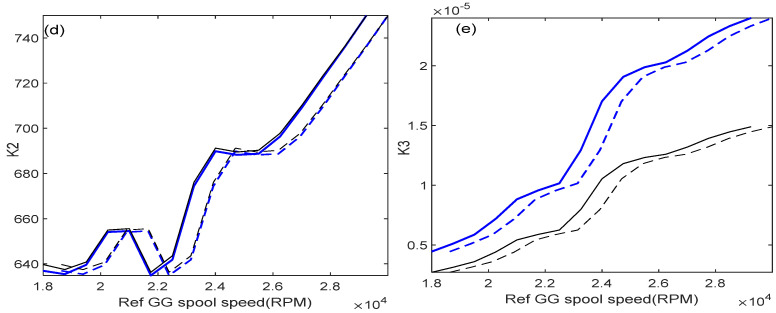
Gain scheduling comparison between diesel No. 1 and bioethanol for unoptimized gain, *a* = 1 (**a**) *K_p_*, (**b**) *K_i_*, (**c**) *K*_1_, (**d**) *K*_2_, (**e**) *K*_3_.

This section investigates the effect of using the bioethanol on the proposed gain scheduling control system. Figure 21 shows the results of the scheduling of the gains of *K_p_*, *K_i_*, *K*_1_, *K*_2_, and *K*_3_. It shows that *K*_3_ indicates a significant difference between the diesel No. 1 and bioethanol. This is because the value of *K*_3_ depends on the value of the fuel flow, ${\dot{m}}_{f}$, where for the bioethanol, the mass flow has significantly increased, as shown in the fuel boundary graph.

The meta-heuristic optimizations have been successfully implemented to solve the gain scheduling optimization of designing a small GT based on the thermodynamics model. The results show that the WOA has better performance than that of the GA, as it exhibits the lowest fitness value. Step response analysis has also shown that the WOA reduces the time to achieve the PLA target significantly, as compare with the unoptimized gain scheduling. The gain scheduling can be used as a guide to select the appropriate value of constant gains; however, it is still a challenge to avoid the oscillation or unstable system response. Thus, for future research, this condition needs to be studied more extensively. Exploring the advance computational strategies to enhance the performance of the meta-heuristic optimizations to optimize the proposed GT engine control system is also necessary.

In general, using bioethanol as the fuel for the designed 1.5 MW GT engine, the mass flow increased. This is due to the low heating value of the bioethanol. The fuel boundaries and gain scheduling are observed, considering the same parameter values of the thermodynamics-based dynamic model. Investigation of the design performance, considering the design point and off-design adaptations, can be considered for future research. The computational success of the off-design performance and gain scheduling can be a positive indication that creation of a renewable energy-based GT engine is possible in the future. Fuel system modification, which may be necessary for the biofuel delivering system, as requested by the control system, should be studied. The implementation of the other meta-heuristic optimizations that exhibit better performance than the proposed method to optimize the renewable fuel-based GT engine is also an important future research field.

## 6. Conclusions

The meta-heuristic optimization was successfully applied to optimize the comprehensive control system of the design of a small GT engine based on the thermodynamics model. The control system consisted of the gain PI controller, with additional gains and gain scheduling and a min-max control strategy, considering fuel limiters. The optimization was conducted through optimizing the additional gain, *K*_3_, based on ITAE as the performance index. The WOA showed better performance than that of the GA, as the WOA resulted in the minimum fitness value. As compare with the unoptimized gain, the time to achieve the target PLA was also significantly reduced. Investigating the possibility to use the constant gain, we found that gain scheduling can be used as the guide to select the proper gains values; however, finding the correct value of the constant gain was still an issue. This requires a computational strategy to select the appropriate gains that do not sustain oscillation. Considering bioethanol as the fuel, it was observed that the fuel flow increased significantly due to the heating value of the bioethanol as compared with that of the diesel No. 1 fuel. As a consequence, the scheduling gain *K*_3_, which depended on the fuel flow, also increased.

## Figures and Tables

**Table 1:** LM2500 technical data [37,38].

Parameter	Ref.
Power (kW)	25,060
SFC (kg/kWh)	0.227
Efficiency (%)	37
Exhaust flow rate (kg/s)	70.3
Exhaust temperature (K)	839.15
Pressure ratio	18

**Table 2:** Validation results of LM2500.

Parameter	Ref.	Present Work	% Error (Present Work)	Error [38]
Efficiency (%)	37	36	2.7	2.652
SFC (kg/kWh)	0.227	0.24	5.7	4.435
Exhaust flow rate (kg/s)	70.3	70	0.43	2.612
Exhaust temperature (K)	839.15	819	2.4	3.174

**Table 3:** SGT-100 technical data.

Parameter	Ref.
Power (kW)	5700
Efficiency (%)	33.5
Exhaust flow rate (kg/s)	19.5
Exhaust temperature (K)	817.5
Pressure ratio	14.9

**Table 4:** Validation results of SGT-100.

Parameter	Ref.	Present Work	% Error
Efficiency (%)	33.5	32.4	3.28
Exhaust flow rate (kg/s)	19.5	19.1265	1.915
Exhaust temperature (K)	817.5	817.5032	0.0004

**Table 5:** Input of gas turbine dynamic model.

GT Components	Symbol	Value	Parameter
Inlet	Δ*P_min_*	4 (in H_2_O)	Inlet total pressure loss
	${\dot{m}}_{a}$	7 kg/s	Inlet air mass flow
Compressor	*B_c_*	0.015	Compressor bleed fraction
	*PR_c_*	7	Compressor pressure ratio
	$\eta_{c,poly}$	0.86	Compressor polytropic efficiency
Combustor	$\eta_{b}$	99%	Combustor efficiency
	Δ*P_b_*	0.05	Combustor total pressure loss
	*HV*	43,100 kJ/kg	Fuel heating value
	*V_b_*	0.0117	Combustor volume
Turbine	TIT	1200	GG turbine inlet temperature
	$\eta_{t,\;poly}$	0.92	GG turbine polytropic efficiency
	*N_gg_*	30,000	GG spool speed
	*I_gg_*	0.08	GG polar moment inertia
Power turbine	$\eta_{pt,\;poly}$	0.92	PT polytropic efficiency
	*N_pt_*	27,000 RPM	PT spool speed
	*I_pt_*	0.05 kg m^2^	PT polar moment of inertia
Exhaust	Δ*P_exhaust_*	8 (in H_2_O)	Exhaust total pressure loss
Shaft	$\eta_{mech}$	99%	Mechanical efficiency
Load	$\eta_{gear}$	100%	Gear box efficiency

**Table 6:** Results of GA and WOA.

	Fitness Value	Best Parameter Value
GA	0.5755	*a* = 3.3704
WOA	0.5737	*a* = 3.3594

**Table 7:** Results of GA and WOA, Case 2.

	Fitness Value	Best Parameter Value
GA	79.91	*a* = 26.38
WOA	79.89	*a* = 27.2848

**Table 8:** Design point comparison between diesel No. 1 and bioethanol under the same input parameters in a gas turbine dynamic model.

Parameters	Diesel No. 1	Bioethanol
Shaft power (kW)	1.4994	1.5347
Thermal efficiency	0.2839	0.2853
Specific fuel consumption (kg/kWh)	0.2942	0.4639
Fuel flow (kg/s)	0.1226	0.1978

## Data Availability

Not applicable.
